# Comprehensive Genomic Characterization Analysis of lncRNAs in Cells With Porcine Delta Coronavirus Infection

**DOI:** 10.3389/fmicb.2019.03036

**Published:** 2020-01-28

**Authors:** Junli Liu, Fangfang Wang, Liuyang Du, Juan Li, Tianqi Yu, Yulan Jin, Yan Yan, Jiyong Zhou, Jinyan Gu

**Affiliations:** ^1^MOE International Joint Collaborative Research Laboratory for Animal Health and Food Safety, Institute of Immunology, Nanjing Agricultural University, Nanjing, China; ^2^MOA Key Laboratory of Animal Virology, Department of Veterinary Medicine, Zhejiang University, Hangzhou, China

**Keywords:** PDCoV infection, RNA-seq, lncRNA, TNF, metabolic pathway

## Abstract

Porcine delta coronavirus (PDCoV) is a novel emerging enterocytetropic virus causing diarrhea, vomiting, dehydration, and mortality in suckling piglets. Long non-coding RNAs (lncRNAs) are known to be important regulators during virus infection. Here, we describe a comprehensive transcriptome profile of lncRNA in PDCoV-infected swine testicular (ST) cells. In total, 1,308 annotated and 1,190 novel lncRNA candidate sequences were identified. Gene Ontology (GO) and Kyoto Encyclopedia of Genes and Genomes (KEGG) analysis revealed that these lncRNAs might be involved in numerous biological processes. Clustering analysis of differentially expressed lncRNAs showed that 454 annotated and 376 novel lncRNAs were regulated after PDCoV infection. Furthermore, we constructed a lncRNA-protein-coding gene co-expression interaction network. The KEGG analysis of the co-expressed genes showed that these differentially expressed lncRNAs were enriched in pathways related to metabolism and TNF signaling. Our study provided comprehensive information about lncRNAs that would be a useful resource for studying the pathogenesis of and designing antiviral therapy for PDCoV infection.

## Introduction

Long non-coding RNAs (lncRNAs), which are transcripts larger than 200 nt in length that lack protein-coding ability, have previously been described in mammalian cells (Kapranov et al., 2007; Mattick and Rinn, 2015). Most of them have a structure similar to mRNA; they have a 5′ methylguanosine cap and are usually spliced and polyadenylated at their 3′ termini. Notably, lncRNA expression shows significant cell and tissue specificity (Mercer et al., 2008; Derrien et al., 2012). Emerging evidence shows that non-coding RNAs have a regulatory role in multiple cellular processes, such as genomic imprinting, chromatin modification, and alternative splicing of RNA (Mercer et al., 2009). Moreover, some diseases such as cancer and neurological disorders are also related to the dysregulated expression of lncRNA (Qureshi et al., 2010; Tsai et al., 2011). Numerous studies have been conducted to ascertain their functional role during viral infection. For example, NRAV can promote influenza virus replication and virulence through negatively regulating the initial transcription of varieties of interferon-stimulated genes (ISGs) (Ouyang et al., 2014). lncRNA-ACOD1, named by its neighboring coding gene aconitate decarboxylase 1, significantly reduces virus multiplication by directly interacting with the metabolic enzyme glutamic-oxaloacetic transaminase (Wang et al., 2017). Neat1, one of the lncRNAs induced by HIV-1 infection, is retained in the nucleus and serves as a scaffold for the nuclear paraspeckle substructure. Importantly, Neat1 deficiency enhances HIV-1 replication (Zhang et al., 2013). Although large amounts of data have proved that several lncRNAs are involved in different kinds of virus infection, the mechanisms by which they act are still largely unknown.

Porcine delta coronavirus (PDCoV), a novel emerging pathogenic enterocytetropic virus, was first discovered from the feces of pigs in Hong Kong in 2012. It is an enveloped, single-stranded positive-sense RNA virus. It belongs to the genus *Deltacoronavirus* in the family *Coronaviridae* of the order *Nidovirales* (Woo et al., 2012). The genome length of PDCoV is approximately 25.4 kb. It is similar in structure to other coronaviruses, with shorter non-coding regions (5′-UTR and 3′-UTR) at both terminals. The 3/4 genome from the 5′ end contains two overlapping open reading frames, ORF1a and ORF1b, encoding the pp1a and pp1b, respectively. The downstream of the genome encodes structural protein spike (S), envelope (E), membrane (M), accessory proteins NS6, structural protein nucleocapsid (N), and accessory proteins NS7 and NS7a. A total of 15 non-structural proteins are encoded by the 5′ terminal of the genome (Fang et al., 2017). PDCoV mainly causes acute, watery diarrhea, vomiting, dehydration, and mortality in suckling piglets, including lesions in the stomach and lungs (Ma et al., 2015). The first outbreak of PDCoV infection was reported in the United States in early 2014 and, to date, it has been detected in Canada, South Korea, China, Thailand, and Vietnam, thus posing huge threat to the swine industry and attracting a great deal of attention (Lee and Lee, 2014; Dong et al., 2016; Lorsirigool et al., 2017; Ajayi et al., 2018; Saeng-Chuto et al., 2019).

During infection, the accessory and non-structural proteins of PDCoV usually perform multiple functions to promote replication in infected cells. A previous study showed that NS6 interaction with RIG-I/MDA5 attenuated the binding activity between RIG-I/MDA5 and double-stranded RNA, resulting in a reduced level of IFN-β production (Fang et al., 2018). Also, the non-structural protein nsp5, a 3C-like protease, is an important molecule in suppressing type I IFN signaling (Zhu et al., 2017b). In addition, NEMO, an essential modulator of NF-κB, can also be cleaved by nsp5, causing inhibition of IFN-β production (Zhu et al., 2017a). Though there are many reports of immune evasion by PDCoV, the precise pathogenic mechanism of PDCoV is largely unclear.

Based on the increasing number of reports on host lncRNAs associated with virus infection, we are interested in investigating whether host lncRNAs were involved in PDCoV infection. In this study, genome-wide profiling of lncRNAs in swine testicular (ST) cells infected with PDCoV was performed using RNA-seq. We identified 830 differentially expressed lncRNAs from PDCoV-infected cells. An integrative analysis of lncRNA alterations suggested their putative role in regulating the expression of several key genes in metabolic and TNF signaling pathways during infection. In conclusion, this work supports the role of lncRNAs as important regulators of PDCoV infection.

## Materials and Methods

### Cells and Viruses

Swine testicular cells and porcine jejunum intestinal epithelial cells (IPEC-J2) were grown in DMEM supplemented with 10% (vol/vol) FBS (Gibco, Carlsbad, CA, United States) at 37°C in a humidified 5% CO_2_ atmosphere. The PDCoV-CH-HA3-2017 (MK040455) strain, stored in our laboratory, was propagated in ST cells.

### Viral Infection and RNA Extraction

For RNA-seq, ST cells were infected with PDCoV at a multiplicity of infection (MOI) of 10; the medium for PDCoV infection was DMEM containing 0.2 ug/ml Trypsin that had been TPCK-treated (Millipore Sigma, St. Louis, MO, United States) for 11 h. Mock-infected cells were placed in the same volume of DMEM, with the same concentration of TPCK-treated Trypsin. Total RNA was isolated from each group using SuPerfecTRI^TM^ Total RNA Isolation Reagent (Pufei, Shanghai, China) according to the manufacturer’s instructions. The RNA quality was checked by 1% agarose gel electrophoresis. The purity and concentration of RNA were measured by NanoPhotometer^®^ spectrophotometer (IMPLEN, München, Germany) and Qubit^®^ RNA Assay Kit in Qubit^®^ 2.0 Fluorometer (Life Technologies, Camarillo, CA, United States). RNA integrity was assessed using the RNA Nano6000 Assay Kit of the Bioanalyzer 2100 system (Agilent Technologies, Santa Clara, CA, United States). For quantitative RT-PCR (RT-qPCR), ST and IPEC-J2 cells were infected or mock-infected with PDCoV at an MOI of 10 and harvested at the indicated time. All experiments were conducted in triplicate.

### RNA-Seq and Data Analysis

Sequencing libraries were generated using the rRNA-depleted RNA with a NEBNext^®^ Ultra^TM^ Directional RNA Library Prep Kit (New England Biolabs, Ipswich, MA, United States). After determining the quality of the library, RNA-seq was performed using an Illumina HiSeq^TM^ 4000 (Illumina, San Diego, CA, United States) to generate raw reads. After removing poly-N sequences, adapters, and low-quality reads, clean reads were obtained and the paired-end reads were aligned to Ensemble pig genome (Release 76). lncRNAs were identified using TopHat2 (v2.0.9), and reads that were mapped to the pig genome were assembled using Cufflinks v2.1.1 (Trapnell et al., 2010). Cuffdiff (v2.1.1) was used to calculate the FPKMs of both lncRNAs and coding genes in each sample. Gene FPKMs were computed by summing the FPKMs of transcripts in each gene group, and differentially expressed (DE) transcripts were assigned where there was a statistically significant level of expression (*p* < 0.05). RNA-seq and data analysis were completed by Novogene.

### GO and KEGG Enrichment Analysis

Gene Ontology (GO) enrichment analysis of differentially expressed genes or lncRNA target genes was conducted with respect to biological process, molecular function, and cellular component with the GOseq R package, in which gene length bias was corrected. Kyoto Encyclopedia of Genes and Genomes (KEGG) was used to perform pathway enrichment analysis^1^. KOBAS software was used to test the level of statistical significance of enrichment of differentially expressed genes and/or lncRNA target genes in KEGG pathways (Mao et al., 2005).

### RT-qPCR, RT-PCR, and Statistical Analysis

To determine the reliability of the RNA-seq data, 15 differentially expressed lncRNAs were randomly selected to test the expression by RT-qPCR. Total RNA was extracted from ST and IPEC-J2 cells using SuPerfecTRI^TM^ Total RNA Isolation Reagent (Pufei, Shanghai, China). First-strand cDNA was synthesized with a reverse transcriptase kit (Vazyme, Nanjing, China). RT-qPCR was performed with a SYBR Green master mix (Vazyme, Nanjing, China) on the LightCycler 96 (Roche, Basel, Switzerland). The PDCoV M gene was detected by RT-PCR. All the primers are presented in Table 1. Relative expressions were calculated using the 2^–ΔΔCt^ method with *GAPDH* as the internal control. Comparisons between groups were made using two-way ANOVA. The data reported are the mean ± SEM. Differences were considered statistically significant when *p* < 0.05.

**TABLE 1:** Primers used for RT-qPCR validation.

**Primer**	**Sequence (5′-3′)**	**Amplicon**
LNC_000034-F	AAGAAAGCGGCAGCCGTGAG	125 bp
LNC_000034-R	TTAATTATTCTCCCTCCGCGTGC	
LNC_000384-F	GCACCCGTCCCCTTCTTTC	166 bp
LNC_000384-R	CCACACGGTCCCCACTTATTC	
LNC_000553-F	TGTGAAGGTCAACTATCTGGGAGC	147 bp
LNC_000553-R	ACACGGGTGAGCTTGGAAATG	
LNC_000597-F	ACAAGCCTTGCCATCATCAAGC	194 bp
LNC_000597-R	CCGAAGTGTCTCTGTATGGAGCTG	
LNC_000625-F	AGTGCCATAGAAGGCGTATTGCAC	180 bp
LNC_000625-R	GTGAATGATGCTGCTTTGAACCTGT	
LNC_000626-F	CACCAAGGCTAAATTCCCAGGTTAC	195 bp
LNC_000626-R	AATACGCCTTCTATGGCACTCACC	
LNC_000660-F	CTCTAAGCATCCGCCACCC	151 bp
LNC_000660-R	TGCCCACCAATCTGTAAGCACTA	
LNC_000819-F	CGCTTGGGTTGCTGTAATGG	114 bp
LNC_000819-R	GGGGAAAGGCGGAGGACTAA	
LNC_000266-F	CAAACGCAGAACACCTGATGTTTG	165 bp
LNC_000266-R	ACGTTTCTAGGGCAGGAGGGAC	
LNC_000676-F	GGGCGGCTGTGGAAGATCAT	101 bp
LNC_000676-R	CCAGAGTCACTGGCTCCAAACAC	
GAPDH-F	TGGTGAAGGTCGGAGTGAAC	225 bp
GAPDH-R	GGAAGATGGTGATGGGATTTC	
PDCoV-M-F	ATGCCACGCGTAATCGTGTGATC	186 bp
PDCoV-M-R	GAGTCATACCAGTACTTGGCCCAGG	

### Construction of the lncRNA-Protein-Coding Gene Co-expression Network

For each lncRNA, the Pearson correlation coefficient of its expression value with that of each protein-coding gene was calculated. Under the conditions of an absolute value of the Pearson correlation coefficient >0.998 and *p* < 0.00001, the interaction network of the differentially expressed lncRNAs and protein-coding gene co-expression pairs was then constructed using Cytoscape (v3.5.1) (Shannon et al., 2003).

## Results

### RNA-Seq and lncRNA Screening in PDCoV-Infected Cells

**FIGURE 1:**
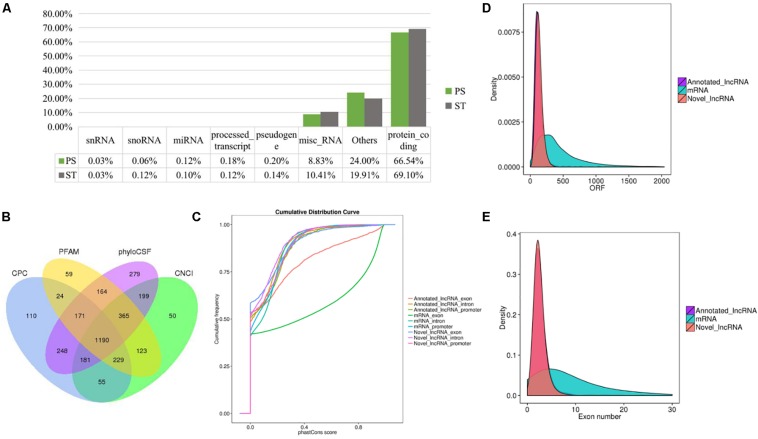
RNA-seq and the characteristics of lncRNAs in PDCoV-infected ST cells. **(A)** RNA categories of quantified genes in RNA-seq. The percentages of each RNA species are shown in infected or mock-infected ST cells. **(B)** Venn diagrams of the identified lncRNAs shared among four software products. CNCI, CPC, PhyloCSF, and PFAM were used to screen novel lncRNAs. The sum of the numbers in each large circle represents the total number of lncRNA candidates of the indicated software, and the overlapping portions of the circles represented the lncRNAs shared between the software products. The 1190 novel lncRNAs were totally identified in ST cells. **(C–E)** Comparison of the conservation **(C)**, exon number **(D)**, and ORF length **(E)** of lncRNAs and protein-coding genes.

To identify the lncRNAs in PDCoV-infected cells, we sequenced the transcriptomes of the ST cells with or without PDCoV infection using high-throughput RNA sequencing. Robust and reproducible data were obtained from all samples, and more than 1 × 10^8^ clean reads per sample were retained after removing reads containing adapter or poly-N sequences and reads with low quality. Afterward, all clean reads were aligned onto the pig reference genome (Release 76) using TopHat2 and were compared and assembled with Cuffcompare and Cufflinks, respectively, and coverage analysis was performed on those clean reads on different annotated gene types. The distribution of each type of gene was counted according to the expression level. In total, eight categories of RNA were identified, according to database annotation of those transcripts, in which the protein-coding genes were highly represented (66.54% in PS and 69.10% in ST, respectively) (Figure 1A). Next, four software tools, CNCI, CPC, PhyloCSF, and PFAM, were used to calculate the protein-coding potential of assembled-transcripts to screen lncRNAs, then taking the intersection of transcripts with no coding potential in these software products as the novel lncRNA (Figure 1B). In total, 1,308 annotated and 1,190 novel lncRNA candidates were identified (Supplementary Tables S1, S2).

It has been reported that lncRNAs, in comparison with protein-coding genes, usually share some common genomic features to their sequences. They are generally shorter in length, have fewer but longer exons, and there is lower evolutionary sequence conservation, with only ∼15% of mouse lncRNAs having homologs in humans. lncRNAs also demonstrate low expression levels (the median is ∼10% of that of protein-coding genes) (Heward and Lindsay, 2014). To further determine the characteristics of the lncRNAs identified in the present study, we compared the transcript length, exon number, and degree of conservation between protein-coding genes and lncRNAs. Conservation analysis of exons, introns, and promoters of lncRNAs and protein-coding genes showed that the exons of protein-coding genes were the most conserved and the exons of lncRNA were far less conserved (Figure 1C). Furthermore, fewer exons and shorter ORFs were found in lncRNAs, which was also consistent with the reported lncRNAs (Figures 1D,E).

### Whole Transcriptome-Wide Functional Prediction of lncRNAs in PDCoV-Infected Cells

**FIGURE 2:**
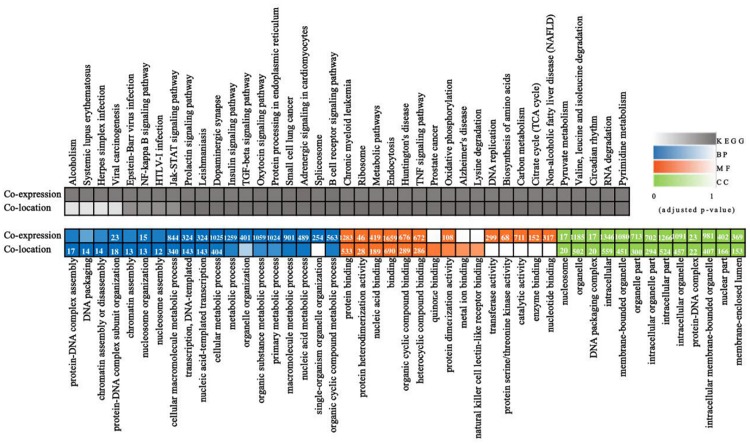
Whole transcriptome-wide functional prediction of lncRNA identified in ST cells. Representative overrepresented KEGG (top) and GO (bottom) terms of gene-expression clusters. The number of genes within each cluster is shown for significantly enriched terms (*p* < 0.05). BP, biological process; MF, molecular function; CC, cellular component.

Long non-coding RNAs sequences are poorly conserved and do not appear to form large homologous families, so it is difficult to infer their common ancestors by sequence similarity (Ponting et al., 2009). Therefore, it is challenging to predict the functions of a type of lncRNA on the basis of its sequence or structure. There have been reports of using genome-wide association analysis between lncRNAs and the co-expressed and/or co-regulated protein-coding genes to characterize the function of the lncRNA (Huarte et al., 2010). To investigate the putative role of lncRNAs, we first analyzed the whole RNA-seq profiles to identify target protein-coding genes whose location or expression was significantly correlated with the candidate lncRNA. For co-located target gene prediction, we searched coding regions 100 k upstream and downstream of lncRNA. In total, 8,812 pairs of lncRNA-protein-coding genes, containing 2,088 lncRNAs and 3,566 protein-coding genes, were identified (Supplementary Table S3). For co-expressed target gene prediction, the expression correlation between lncRNAs and protein-coding genes was evaluated. When the required Pearson correlation coefficient was set above 0.95, 1,048,575 pairs of lncRNA-protein-coding genes, containing 1,730 lncRNAs and 10,581 protein-coding genes, were obtained (Supplementary Table S4). We next performed GO and KEGG pathway analysis for the target genes of lncRNAs. The top 20 GO and KEGG pathways with the highest representation of each term are reported (Figure 2 and Supplementary Tables S5, S6). KEGG enrichment analysis revealed that pathways related to the immune system and metabolism were preferentially targeted. The GO-term analysis was divided into three main categories: cellular component, biological process, and molecular function. Significantly, a large number of biological processes, like protein-DNA complex assembly, DNA packaging and transcription, and the cellular macromolecule metabolic process, were enriched. Furthermore, protein binding and nucleic acid binding and the nucleosome and organelles, belonging to molecular function and cellular component, respectively, were also enriched. GO and KEGG pathway enrichment analysis of target genes revealed that lncRNAs may act in *cis* or *trans* to participate in the regulation of expression of multiple important genes in different processes including protein binding, DNA transcription, metabolism, and immune response.

### Clustering Analysis Identified Differentially Expressed lncRNAs in PDCoV-Infected Cells

**FIGURE 3:**
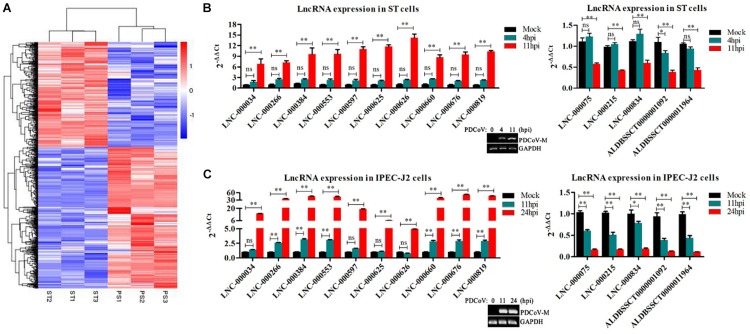
Identification of differentially expressed lncRNAs during PDCoV infection. **(A)** Hierarchic clustering analyses of 830 lncRNAs that were differentially expressed in the PDCoV-infected ST cells (PS) compared with the mock-infected ST cells (ST) (*p* < 0.05). **(B,C)** Validation of 10 up-regulated (left) and five down-regulated (right) lncRNAs from RNA-Seq in ST **(B)** and IPEC-J2 cells **(C)** were performed by RT-qPCR. The PDCoV M gene was confirmed by RT-PCR. Data are shown as mean ± SEM, *n* = 3. ^∗^*p* < 0.05, ^∗∗^*p* < 0.01.

To identify the PDCoV-associated lncRNAs, Cuffdiff software was used to investigate the differentially expressed (DE) lncRNAs in PDCoV-infected cells. The hierarchical clustering heat map in Figure 3A shows the DE lncRNA expression profiling data. Out of the 1,308 annotated and 1,190 novel lncRNAs, we obtained 454 annotated DE lncRNAs (225 up-regulated and 229 down-regulated) and 376 novel DE lncRNAs (252 up-regulated and 124 down-regulated) after PDCoV infection (*p* < 0.05; Supplementary Table S7). Importantly, we observed 20 lncRNAs whose expression levels were decreased to FPKM = 0 after PDCoV infection, while the FPKMs of another 12 lncRNAs, all novel lncRNAs, were 0 before PDCoV infection (Supplementary Table S7). This suggests that these 32 lncRNAs, though they have very low expression levels, might be strongly associated with the viral infection. Furthermore, to evaluate the reliability of RNA-seq data analysis, 15 lncRNAs were selected for RT-qPCR analysis in PDCoV-infected cells. As shown in Figure 3B, the expression levels of the 15 selected lncRNAs, though exhibiting no significant differences at 4 h post-infection (hpi), were all significantly changed at 11 hpi in ST cells. Also, different expression patterns of lncRNAs were detected in IPEC-J2 cells. As shown in Figure 3C, 11 out of the 15 selected RNA were significantly altered at 11 hpi, and all of them were differently expressed at 24 hpi. For both ST and IPEC-J2 cells, they had a strong expression pattern consistent with the RNA-seq results (Table 2).

**TABLE 2:** Expression level of 15 selected lncRNAs in RNA-seq.

**Transcript_id**	**PS_FPKM**	**ST_FPKM**	**Log2 (fold change)**	***P* value**
LNC_000034	686.329	0	inf	5.00E−05
LNC_000266	2.36983	0.0858038	4.7876	0.00615
LNC_000384	1.31737	0	inf	5.00E−05
LNC_000553	1.39484	0	inf	5.00E−05
LNC_000597	1.38574	0.0480706	4.84936	0.0001
LNC_000625	6.10358	0.199175	4.93754	5.00E−05
LNC_000626	10.5832	0.256783	5.36509	5.00E−05
LNC_000660	3.55981	0.024732	7.16928	0.01105
LNC_000676	0.925249	0.0338341	4.77329	0.005
LNC_000819	1.44203	0	inf	5.00E−05
ALDBSSCT0000001092	0.442178	3.44013	–2.95976	5.00E−05
ALDBSSCT0000011964	0.0812138	0.613515	–2.9173	5.00E−05
LNC_000075	0.221325	1.10022	–2.31355	0.00015
LNC_000215	0.194968	1.04174	–2.41769	5.00E−05
LNC_000834	0.126095	1.51747	–3.58908	0.00145

### Co-location Analysis of DE lncRNAs Revealed Their Potential Regulation of Their Neighboring Protein-Coding Genes

**FIGURE 4:**
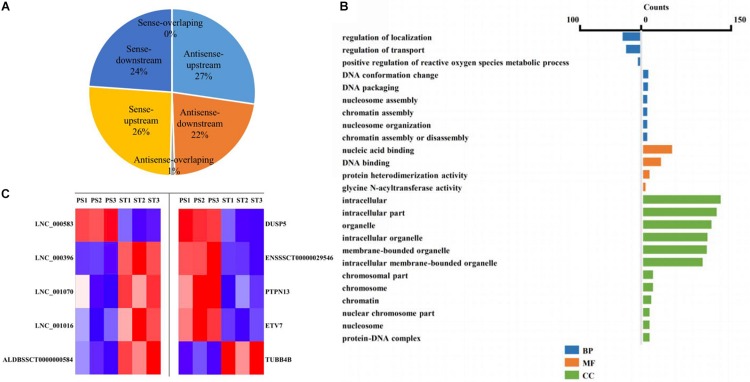
Co-location analysis revealed the potential regulation ability of lncRNAs on their neighboring protein-coding genes. **(A)** Pie chart showing the percentage of each class of lncRNA classified by their location. **(B)** Over-represented GO terms of up-regulated (left) and down-regulated (right) co-located genes (*p* < 0.05). BP, biological process; MF, molecular function; CC, cellular component. **(C)** Heatmap showing the expression correlation of selected antisense lncRNA-protein-coding gene pairs.

The lncRNA in the genome is not randomly distributed, so locus classification will be an effective first step in analyzing its regulatory functions at the genome level (Luo et al., 2016). In general, lncRNAs function either in *cis* or in *trans* to affect the transcription of genes within or far from the same genomic locus (Clark and Blackshaw, 2014). To understand the potential functional association between lncRNAs and cognate genes, we investigated their genomic distribution pattern relative to protein-coding loci and classified all DE lncRNAs to ascertain their potential biological roles. All DE lncRNAs were classified into six categories comprising sense-upstream lncRNA, sense-downstream lncRNA, sense-overlapping lncRNA, antisense-upstream lncRNA, antisense-downstream lncRNA and antisense-overlapping lncRNA. As shown in Figure 4A, 26% of DE lncRNAs were located in the same strand but upstream of protein-coding genes and 24% were located downstream, while antisense-upstream and antisense-overlapping comprised 27 and 1%, respectively, and the remaining 22% were antisense-downstream lncRNAs. Next, in order to define the lncRNA functions more precisely, GO enrichment analysis of the co-located genes of up- and down-regulated lncRNAs were analyzed independently. The results showed that protein-coding genes associated with DE lncRNAs were mainly enriched in terms of molecular function and cellular component, primarily under the category of nucleic acid binding and intracellular membrane-bounded organelle (Figure 4B). Notably, by analyzing the relative expression level, we found that antisense lncRNA and protein-coding genes were specifically co-expressed, in which two pairs showed a positive and three pairs showed a negative correlation in their expression patterns (Figure 4C and Supplementary Table S8). We speculated that these antisense lncRNAs act in *cis* to modulate the expression of their cognate genes.

### Correlation Analysis Provided a Resource to Functionally Identify PDCoV Driver Metabolism- and Immunity-Related lncRNAs

**FIGURE 5:**
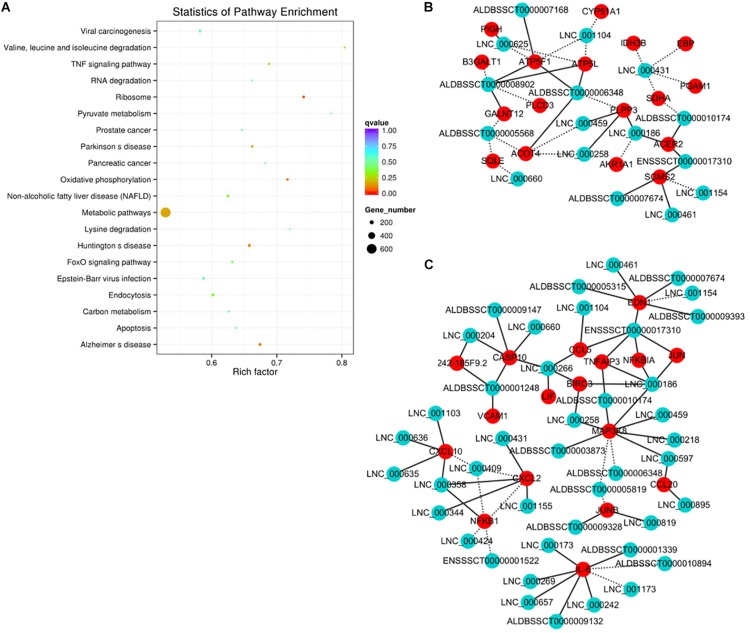
Correlation analysis provides a resource to functionally identify PDCoV driver metabolism- and immunity-related lncRNAs. **(A)** The top 20 overrepresented KEGG pathways of co-expressed genes. **(B,C)** The network of co-expressed lncRNA-protein-coding gene pairs based on a threshold of a Pearson’s correlation coefficient of 0.998. The network of lncRNAs with metabolic- **(B)** or immunity-related genes **(C)** is presented (*p* < 0.00001). The solid line represents a positive correlation, while the dotted line represents a negative correlation.

The functional association between regulatory lncRNA and protein-coding gene transcripts can be determined by performing expression correlation analysis coupled with ascertaining their putative role in related physiological processes. To further investigate the potential mechanism of action of the PDCoV-associated lncRNAs, the DE lncRNAs and their predicted target DE protein-coding genes were investigated by delineating lncRNA-protein-coding gene functional interactions. Here we identified 1,048,575 pairs of DE lncRNA-DE protein-coding genes, containing 821 lncRNAs and 8,799 protein-coding genes (*p* < 0.01). Next, KEGG pathway analysis was repeated once again (Figure 5A), and we found that metabolic and TNF signaling pathways were significantly enriched.

The interaction network involving the metabolic and TNF signaling pathways was then constructed. Several key genes in metabolism were positively or negatively regulated by lncRNAs (Figure 5B). Of the significantly enriched genes, ATP5L and ATP5F1, two of the mitochondrial membrane ATP synthase subunits, were regulated by LNC-000625, LNC-001104, ALDBSSCT0000008902, and ALDBSSCT0000006348. In addition, three lncRNAs, LNC-000459, LNC-000258, and ALDBSSCT0000005568, might regulate acyl-coenzyme A thioesterase four expression. These results suggest that these lncRNAs might be involved in the regulation of metabolic processes particularly involving energy and lipid metabolism. Meanwhile, an inducible program of inflammatory gene expression is central to antiviral defense. Many of them, i.e., CCL5, CCL20, CXCL2, CXCL10, MAP3K8, NF-κB1, and interleukin 6 (IL-6), were protein-coding genes known to have roles in the inflammatory response. In the network (Figure 5C), eight lncRNAs have putative regulatory roles in IL-6 expression. Six of them, LNC-000173, LNC-000269, LNC-000242, LNC-000657, ALDBSSCT0000009132, and ALDBSSCT0000001339, might exert positive regulation, while LNC-001173 and ALDBSSCT0000010894 showed the opposite effect. This suggests that these lncRNAs might act as the regulatory module of the circuit that is involved in the inflammatory response.

## Discussion

Numerous studies have shown that lncRNAs play a key role during viral infection. The lncRNAs THRIL, NeST, NEAT, and lincRNA-Cox2 can participate in immune responses against viral infection mainly through regulating the expression of TNF-α, IFN-γ, IL8, and inflammatory response, respectively (Carpenter et al., 2013; Gomez et al., 2013; Imamura et al., 2014; Li et al., 2014). PDCoV is an important enteric virus mainly causing diarrhea in suckling pigs. Infection with PDCoV causes changes in the expression levels of several host cell proteins of host innate immune response, but little is known about the critical roles of lncRNAs in these processes.

Here, we performed RNA-seq to identify the lncRNAs involved in PDCoV infection. The results of comparing clean reads to the genome showed that more than 60% of reads are protein-coding genes, and no lncRNA classifications were identified due to the limited lncRNA database annotation in pig. In our results, 1,190 novel lncRNAs were identified. Further analysis showed that the basic characteristics of these novel lncRNAs are consistent with the known ones. Our RNA-seq results further enrich the pig lncRNA database.

In total, we found 454 annotated and 376 novel lncRNAs that were differentially expressed during PDCoV infection. These lncRNAs were classified as sense-upstream lncRNA, sense-downstream lncRNA, sense-overlapping lncRNA, antisense-upstream lncRNA, antisense-downstream lncRNA, and antisense-overlapping lncRNA. Many antisense-overlapping lncRNAs have inverse expression patterns with their sense transcript counterparts. This suggests that these antisense-overlapping lncRNAs may have a negative regulatory effect on them. In contrast, many lncRNAs that do not contain overlapping sequences display expression patterns correlated with their neighboring protein-coding gene transcripts. In the present study, two out of five antisense overlapping lncRNAs were found to have high consistency in their expression (Figure 4). Similarly, the lncRNA *Evx1as*, which initiates within the first exon of the gene *EVX1*, has an overlap of eight nucleotides with the EVX1 mRNA and promotes transcription of its neighbor gene by increasing the binding affinity of histone H3 lysine 4 tri-methylation (H3K4me3) and histone H3 lysine 4 acetylation (H3K27ac) at the promoter region. Considering that most lncRNAs might function through their secondary structure rather than the primary one, this suggests that the regulation of antisense transcripts by antisense-overlapping lncRNA may not be mediated through base-complementary pairing.

Correlation analysis of DE lncRNA and protein-coding genes identified a number of DE lncRNA-DE protein-coding gene pairs. The main enriched KEGG pathways of these protein-coding genes were in metabolism and oxidative phosphorylation. In a recent report, 5-day-old neonatal pigs were infected with PDCoV, and transcriptome profile and KEGG pathway enrichment analysis were performed at different stages of infection (Wu et al., 2019). In our study, we found that the lncRNA targeted genes enriched in those pathways that were perturbed during the late stage of infection. In addition, the expression level of transglutaminase 3 (TGM3) and apolipoprotein A-2 (APOA2) in a Wu et al. (2019) study were significantly changed. Similarly, we also found that TGM1 was up-regulated, and APOA1, APOA4, and APOA5 were down-regulated during PDCoV infection (data not shown). Moreover, our data show that many cytokines and chemokines, which elicit an inflammatory response, were differentially expressed in the infected cells compared to mock cells. The inflammation causes injury to the intestinal tissues, resulting in diarrhea or even death. Raised CCL and CXCL10 levels were associated with the severity of virus infection (Betakova et al., 2017; Masood et al., 2018). Here, we identified a number of lncRNAs that may regulate the expression of these inflammatory molecules.

To the best of our knowledge, this is the first study focusing on the expression profile of cellular lncRNAs after PDCoV infection. Our data show the expression landscape of lncRNAs, with special emphasis on the lncRNA-protein modules operating in response to PDCoV infection. Moreover, this study provides a comprehensive genome-wide resource for exploring the molecular and cellular regulatory functions of lncRNAs. This study will also be useful for identifying lncRNAs as potential biomarkers for the diagnosis of PDCoV infection and designing better prophylactic and therapeutic tools against virus infection.

## Conclusion

In the present study, the expression profiles of lncRNAs were determined in PDCoV-infected ST cells. In total, 1,190 novel lncRNAs were identified. A total of 830 lncRNAs were differentially expressed between PDCoV-infected or mocked-infected ST cells. KEGG pathway analysis of DE lncRNA co-expressed genes revealed that they might be primarily involved in regulating metabolism and TNF signaling pathways. Our study systematically characterizes lncRNA expression during PDCoV infection and provides a useful resource for identifying and functionally characterizing the cognate gene products of those lncRNAs. This study will also be useful for assigning lncRNAs as potential biomarkers of PDCoV infection and designing better preventive and therapeutic measures against the virus infection, which would be economically beneficial for the pig farming community.

## Data Availability Statement

The raw data supporting the conclusions of this article will be made available by the authors, without undue reservation, to any qualified researcher.

## Author Contributions

JLL, JG, and JZ conceived and designed the experiments. FW, LD, and JL performed the experiments. JLL, YY, YJ, and TY analyzed the data. JLL drafted the manuscript. All authors read and approved the final manuscript.

## Conflict of Interest

The authors declare that the research was conducted in the absence of any commercial or financial relationships that could be construed as a potential conflict of interest.

## Footnotes

1 http://www.genome.jp/kegg/
